# Association of dietary patterns with the fecal microbiota in Korean adolescents

**DOI:** 10.1186/s40795-016-0125-z

**Published:** 2017-03-04

**Authors:** Han Byul Jang, Min-Kyu Choi, Jae Heon Kang, Sang Ick Park, Hye-Ja Lee

**Affiliations:** 1 Center for Biomedical Science, Korea National Institute of Health, Cheongju, Chungcheongbuk-do Republic of Korea; 2 Department of Family Medicine, Gangnam Hallym Sacred Heart Hospital, Hallym University, Seoul, Republic of Korea; 3 Department of Family Medicine, Obesity Research Institute, Seoul Paik Hospital, College of Medicine, Inje University, Seoul, Republic of Korea

**Keywords:** Dietary patterns, Gut microbiota, Metabolic syndrome

## Abstract

**Background:**

The gut microbiota has emerged as an important environmental factor associated with obesity, type 2 diabetes, and cardiovascular disease, through its interactions with dietary factors. Therefore, we analyzed the composition of the fecal microbiota and levels of biochemical markers related to metabolic disease according to dietary pattern in Korean adolescents.

**Methods:**

We collected fecal samples from 112 student subjects aged 13–16 years with sufficient information available regarding clinical biomarkers and diet, and performed 16S rRNA targeted gene sequencing.

**Results:**

Regarding bacterial composition according to taxonomic rank, we found that traditional dietary patterns enriched in plant-based and fermented foods were associated with higher proportions of *Bacteroides* (*Bacteroidaceae*) and *Bifidobacterium* (*Bifidobacteriaceae*-*Actinobacteria*) and a lower proportion of *Prevotella* (*Prevotellaceae*) relative to modified Western dietary patterns (a greater proportion of animal-based foods). Specifically, the proportion of *Bacteroides* (*Bacteroidaceae*) was associated with intake of plant-based nutrients such as fiber; however, that of *Prevotella* (*Prevotellaceae*) was negatively associated with these factors. Additionally, we observed that the increase of *prevotella* (*Prevotellaceae*) and decrease of *Bacteroides* (*Bacteroidaceae*) and *Ruminococcaceae* had a higher risk of obesity. We also found that the traditional dietary pattern was negatively associated with general and central adiposity and levels of clinical biomarkers, including AST, ALT, total cholesterol, triglyceride, hs-CRP, insulin, and HOMA-IR, whereas the positive associations were found for a modified Western dietary pattern.

**Conclusions:**

These findings suggest that the gut microbiota composition differs markedly according to dietary intake and suggest a role for diet in promoting a gut microbiome associated with the pathogenesis of metabolic disease.

**Electronic supplementary material:**

The online version of this article (doi:10.1186/s40795-016-0125-z) contains supplementary material, which is available to authorized users.

## Background

The increased prevalence of childhood obesity worldwide is associated with serious health risks, such as insulin resistance (IR), type two diabetes (T2D), and cardiovascular disease (CVD) in childhood and later life [1]. The etiology of obesity and its metabolic consequences are complex and involve environmental factors that are challenging to modify. It is therefore crucial to identify modifiable risk factors involved in the early development of metabolic disorders to facilitate prevention and treatment.

In recent years, the gut microbiota has emerged as an important environmental factor associated with host health. The gut is home to trillions of microbes, and plays a major role in energy metabolism and the immune system [2, 3]. Several studies in animal models and humans have suggested that the gut microbiota is linked to complex disease phenotypes such as obesity and insulin resistance [4, 5]. A balanced gut microbiota composition confers benefit to the host, whereas microbial dysbiosis is implicated in various diseases, including obesity, T2D, and CVD. These studies imply that the effects of environmental factors on the development of metabolic-related disorders are mediated in part by altered gut microbial composition and function.

Therefore, research into factors affecting the gut microbiota has become an area of growing scientific interest. The gut microbiota is influenced by various factors, including the microbial species acquired at birth, age, host genotype, geography, and diet [6–9]. Of these, diet is considered a key contributor to the diversity of the gut microbiota, explaining 57% of the total structural variation, while only 12% is related to genetic differences [10]. Therefore, many studies have focused on the relationship between the gut microbiota and dietary factors, such as dietary pattern (vegetarian and Western) [11, 12], specific foods (whole grain products, fruits, and vegetables) [13–17], and food constituents (dietary fiber, fat, and protein) [8, 18–20]. Interestingly, a recent study showed that European children who consumed a typical Western diet had a microbiota enriched in *Firmicutes* and *Enterobacteriaceae*, whereas rural African children, who consumed a diet low in fat and animal protein and rich in plant-based foods, had greater abundances of the genera *Bacteroidetes* and *Prevotella* [7]. Another study demonstrated that microbiota composition was strongly associated with long-term diet [8]. *Bacteroides* abundance was found to be associated with a diet enriched in animal products, whereas that of *Prevotella* was correlated with diets that contained more plant-based foods.

Dietary factors can vary widely according to ethnicity and geographical location; however, few studies have focused on Korean populations. Therefore, we performed an in-depth analysis of the association between the fecal microbiota and dietary factors in Korean adolescents. Because dietary intake is a complex exposure variable, we used a total diet approach to identity the overall dietary patterns of Korean adolescents and then examined their associations with the gut microbiota composition and levels of biochemical markers. Assessment of food intake as a whole using dietary pattern analysis yielded useful information regarding the link between diet and the gut microbiota that will facilitate formulation of dietary guidelines to prevent disease.

## Methods

### Study participants

For this study, data were obtained from the Korean Children-Adolescents Study (KoCAS) conducted by the Korea National Institute of Health (KNIH) [4, 21]. A total of 135 volunteers on collection of stool samples, aged 13–16 years, including those with severe obesity, were recruited from Seoul and Kyunggi Province in 2012. After excluding those who did not respond to the dietary survey (*n* = 19) and those for whom insufficient clinical biomarker information was available (*n* = 4), data from 112 subjects were analyzed. The study was approved by the Institutional Review Boards of Seoul-Paik Hospital (IIT-2012-092), Inje University, and the Korea Center for Disease Control (KCDC) and Prevention (2012-04EXP-06-R).

### Anthropometric and biochemical measurements

Professionally trained personnel performed the anthropometric examinations and blood collections using a standardized protocol. The levels of total cholesterol (TC), triglycerides (TG), and HDL cholesterol (HDL-C) were measured via enzymatic assays, and fasting serum glucose levels were measured using the hexokinase method (Autoanalyzer Model 7600 II; Hitachi, Tokyo, Japan). The levels of high-sensitivity C-reactive protein (hs-crp) were measured by latex agglutination turbidimetry and an analyzer (Model 7600; Hitachi, Tokyo, Japan). Fasting serum insulin levels were measured using an enhanced chemiluminescence immunoassay analyzer (E170; Roche, Germany). Insulin resistance (IR) was estimated from fasting serum measurements using the homeostasis model assessment of insulin resistance (HOMA-IR): insulin (μIU/mL) × glucose (mmol/L) ÷ 22.5.

### Isolation of fecal DNA and pyrosequencing of 16S rRNA

The stool samples were self-collected in sterile boxes containing dry ice, transported to the study center with dry ice within 12 h, and stored at −70 °C in the laboratory freezer until DNA extraction. DNA was extracted using a QIAamp DNA Stool kit (Qiagen, Valencia, CA), and the 16S rRNA gene fragments were amplified. The following barcoded primers were designed to target the hypervariable regions (V1 to V3) within the 16S rRNA gene as described previously [4]. The quality of the PCR product was assessed using a Gel Doc system (Bio–Rad, Hercules, CA, USA), and the amplified products were purified using a QIAquick PCR Purification Kit (Qiagen, Valencia, CA, USA). Short DNA fragments were removed using an Ampure Beads Kit (Agencourt Bioanalyzer, MA, USA). The quality and size of the products were assessed using a Bioanalyzer 2100 (Agilent, Palo Alto, CA, USA). The 16S rRNA gene amplicons were sequenced using the GS Junior Sequencing System (Roche, Branford, CT, USA) according to the manufacturer’s protocol. The raw reads were filtered to remove those with a low quality score (average score <25) or reads less than 300 bp. Potential chimeric sequences were detected using the Bellerophon method [22].

### Determination of operational taxonomic units and taxonomic classification

To estimate the taxonomic composition of each sample, the numbers of operational taxonomic units (OTUs) in the filtered reads were calculated. As reported previously [4], the number of OTUs was determined by clustering the sequences from each sample using a 97% sequence identity cutoff [23, 24] using the QIIME software (v. 1.8.0). Taxonomic abundance was assessed using RDP Classifier v. 1.1 with a confidence threshold of 0.8 derived from the pre-processed reads for each sample. The microbial compositions were normalized using the value calculated from the count of taxonomy abundance divided by the number of pre-processed reads of each sample. To normalize the sequence reads, we calculated the extent of coverage using the number of singleton phylotypes and the number of sequences which is a percentage value indicative of how complete sample coverage was. Also, we generated rarefaction curves using a re-sampling without replacement approach to calculate the observed number of OTUs on each iteration. We plotted the curves allowed for comparison of sampling intensity and rarefaction curves appeared parallel. A total of 1,185,358 high-quality sequences (reads) (range, 2,065–42,522 reads) from 135 samples were obtained after filtering out primer sequences and low-quality and chimeric sequences.

### Assessment of dietary intake

Typical dietary intake for each subject was estimated based on 3-day food diaries maintained for three consecutive days (2 weekdays and 1 weekend day), which were completed by 112 adolescent subjects. Dietary questionnaires certified by Inje University Paik Hospital and Hallym University Sacred Heart Hospital were checked by researchers trained to determine whether records contained sufficient information. Nutrient intakes were determined from food intakes using the Computer-Aided Nutritional Analysis for Professionals software v. 3.0 (CAN-pro 3.0, Korean Nutrition Society, Seoul, Korea).

### Dietary pattern analysis

For a factor analysis [25] to generate dietary patterns, foods from the data of the 2012 KoCAS were classified into 31 food groups based on those used in the Korean Nutrient Database [26] and designed to reflect the food habit characteristics of adolescents (Additional file 1: Supplementary Table S1). The factors were rotated using Varimax rotation to achieve a simpler structure with greater interpretability. We considered components with an eigenvalue by scree test > 2 as significant to identify more meaningful factors. To identify the characteristics of the factors, the collection of food groups with an absolute value factor loading of at least 0.2 was used [27]. A cluster analysis was performed using two factor scores for the subjects calculated by factor analysis, and subjects were grouped into two clusters of dietary patterns through the k-means clustering method.

### Definition of metabolic syndrome

Metabolic syndrome was defined as the International Diabetes Federation (IDF) in children and adolescents [28]. According to this criteria, subjects with abdominal obesity (waist circumference above 90th percentile for age, sex and ethnicity), and presenting two or more other clinical features (elevated TG, glucose, blood pressure and low HDL-C), were considered as having metabolic syndrome.

### Statistical analysis

Statistical analysis was performed using the SAS software package (version 9.2; SAS Institute, Inc., Cary, NC, USA), and values are presented as means ± standard deviations (SD) for continuous variables or as raw numbers and percentages for categorical variables. Variables with non-normal distributions were log-transformed before analysis. Microbial alpha diversity of each sample using the Shannon Index with OTUs, *H*′ = − ∑_*i* = 1_^*S*^(*pi* ln(*p*_*i*_)) was measured and Beta diversity was measured by the difference in organism composition between samples using a Bray-Curtis distance $BCij=\frac{Si+ Sj-2 Cij}{Si+ Sj}$. Principal component analysis (PCA) from measured beta diversities was performed in microbiota. Mean values were compared between dietary pattern groups using the general linear model adjusted for age and gender. Chi-squared and Fisher’s exact tests were used to compare prevalence data, and Spearman’s correlation coefficients were used to evaluate the relationship between the abundance of gut microbial taxa and diet. The association between fecal microbiota and metabolic disease were analyzed using a logistic regression model to estimate the odds ratios (ORs) and 95% confidence intervals (CI) after adjusting for age and sex. Differences in the relative abundance of components of the fecal microbiota between the dietary pattern groups were analyzed using the nonparametric Wilcoxon rank-sum test and correction was performed by FDR multiple test. Heat maps and box plots were generated using the R package. *P* values <0.05 and adjusted *P*-values < 0.05 were considered to indicate statistical significance.

## Results

### Dietary pattern

Two major factors were extracted by factor analysis using 31 food groups (Table 1). Factor 1, which had the highest eigenvalue, was characterized as plant-based and fermented foods because it showed higher factor-loading values for whole grains, sweet potatoes, sweets, vegetables, shellfish, seaweeds, and Oriental sources, whereas the contributions of flour, bread, instant products, poultry, eggs, milks, fats, carbonated beverages, and seasonings were negative. Factor 2 was characterized as animal-based foods, with intake of potatoes, nuts & seeds, red meats, fish, oils, fats, and seasonings, but noodles, cookies, fast food, legumes, fruits, milk, and yoghurts had negative contributions. White rice, which is the staple in the Korean diet, had high loadings in both the traditional and modified Western diets.

**Table 1:** Factor loading matrix for the two major dietary patterns

Food groups	Dietary pattern factors	
	Factor1	Factor2
White rice	0.41	0.42
Whole grains	0.35	
Noodle		−0.50
Cereal		
Flour and bread	−0.59	
Cookie, cracker and chip		−0.46
Instant products	−0.47	
Fast Food		−0.36
Potatoes		0.31
Sweet potatoes	0.29	
Sweet	0.32	
Legumes		−0.26
Nuts and Seeds		0.37
Vegetables	0.66	
Mushroom		
Fruits		−0.26
Red meats		0.21
Poultry	−0.42	
Meat products		
Eggs	−0.30	
Fish		0.26
Shellfish	0.25	
Seaweeds	0.28	
Milks and ice cream	−0.30	−0.34
Yoghurt and cheese		−0.28
Oils		0.53
Fats	−0.41	0.37
Carbonated beverage	−0.27	
Beverages		
Oriental sauce	0.72	
Seasonings	−0.24	0.62

Absolute values < 0.2 are not presented in the table for simplicity

To categorize the subjects according to dietary pattern, we performed a cluster analysis based on the two factors and derived two clusters that comprised 73.2 and 26.8% of the total subjects, respectively. Factor scores of the two clusters are presented in Table 2. Cluster 1 had characteristics of a traditional (Korean) dietary pattern, with the highest score for Factor 1. Cluster 2 had a high score for Factor 2, representing a modified Western dietary pattern.

**Table 2:** Classification of subjects by cluster analysis using factor scores

	Cluster1	Cluster2
	(Traditional diet)	(Modified Western diet)
Factor1	0.37 ± 0.67	−1.01 ± 1.06
Factor2	−0.30 ± 0.87	0.82 ± 0.89

Data are expressed as the mean ± S.D

### Nutrient intakes

The nutrient intakes of the two dietary pattern groups are presented in Additional file 1: Supplementary Table S2. Cluster 1 had significantly higher intakes of carbohydrate, plant protein, fiber, vitamin B_6_, folate, vitamin C, phosphorus, potassium, calcium, iron (especially plant iron), and sodium than Cluster 2. Additionally, fat intake in Cluster 1 was significantly lower than that in Cluster 2. The percentage of energy from carbohydrate was higher in Cluster 1, whereas that from fat was higher in Cluster 2.

### Clinical biomarkers according to dietary pattern group

Table 3 summarizes the clinical and biochemical characteristics of the subjects stratified according to dietary pattern group. There were no significant differences in age, sex, height, glucose, and HDL-cholesterol (HDL-C) between the dietary pattern groups. However, Cluster 2 had significantly higher levels of general and central adiposity—including BMI, waist circumference, fat percent, and fat mass—and clinical biomarkers such as liver enzymes (AST and ALT), TC and TG levels, hs-crp, insulin and insulin-resistance surrogate markers (HOMA-IR) compared with Cluster 1. The prevalence of obesity and metabolic syndrome (MS) was also higher in Cluster 2 compared with Cluster 1 (86.7 and 39.0% for obesity (*P* = <0.0001), 17.3 and 36.7% for MS (*P* = 0.0299), respectively).

**Table 3:** General chacteristics of subject according to dietary patten groups

	Traditional diet	Modified Western diet	*P* ^a^
	(*n* = 82)	(*n* = 30)	
Age (years)	13.9 ± 0.6	13.8 ± 0.7	0.2893
Male (n, %)	45 (54.9%)	19 (63.3%)	0.4233^b^
Height (cm)	164.7 ± 7.4	164.8 ± 7.3	0.8236
Weight (kg)	70.9 ± 24.6	90.1 ± 18.2	<0.0001
BMI (kg/m^2^)	25.9 ± 7.9	33.1 ± 5.8	<0.0001
Waist circumference (cm)	82.8 ± 20.3	100.4 ± 15.3	<0.0001
Fat percent (%)	30.9 ± 13.9	42.8 ± 10.7	<0.0001
Fat mass (g)	24.6 ± 17.9	40.0 ± 13.5	<0.0001
AST (IU/L)	23.3 ± 10.7	31.7 ± 18.1	0.0048
ALT (IU/L)	24.6 ± 26.8	47.2 ± 41.6	0.0016
Glucose (mg/dL)	92.6 ± 6.4	94.6 ± 10.3	0.2269
Triglyceride (mg/dL)^c^	99.1 ± 70.3	127.0 ± 63.9	0.0098
Total cholesterol (mg/dL)	163 ± 28.7	179.9 ± 29.1	0.0024
HDL-cholesterol (mg/dL)	50.0 ± 9.5	47.3 ± 10.2	0.1853
hs-CRP^c^	0.14 ± 0.14	0.35 ± 0.44	0.0002
Insulin^c^	17.4 ± 12.8	28.0 ± 31.1	0.0043
HOMA-IR^c^	4.0 ± 3.0	6.7 ± 8.0	0.0039
Obesity (n, %)^d^	32 (39.0%)	26 (86.7%)	<0.0001^b^
Metabolic syndrome (n, %))	14 (17.3%)	11 (36.7%)	0.0299^b^

Data are expressed as the mean ± S.D or n (%)

^a^ *P* value was calculated by generlized linear regression analysis with age, sex and energy intakes

^b^ *P* value was obtained by Chi-squre test

^c^Data are log transformed prior to analysis

^d^Obesity was defined as those with a BMI ≥95th percentile or ≥25 kg/m^2^

*AST*, aspartate aminotransferase; *ALT*, alanine aminotransferase; *hs-CRP*, high-sensitivity C-reactive protein; *HOMA-IR*, homeostasis model assessment of insulin resistance

### Fecal microbial composition according to dietary pattern group

**Fig. 1:**
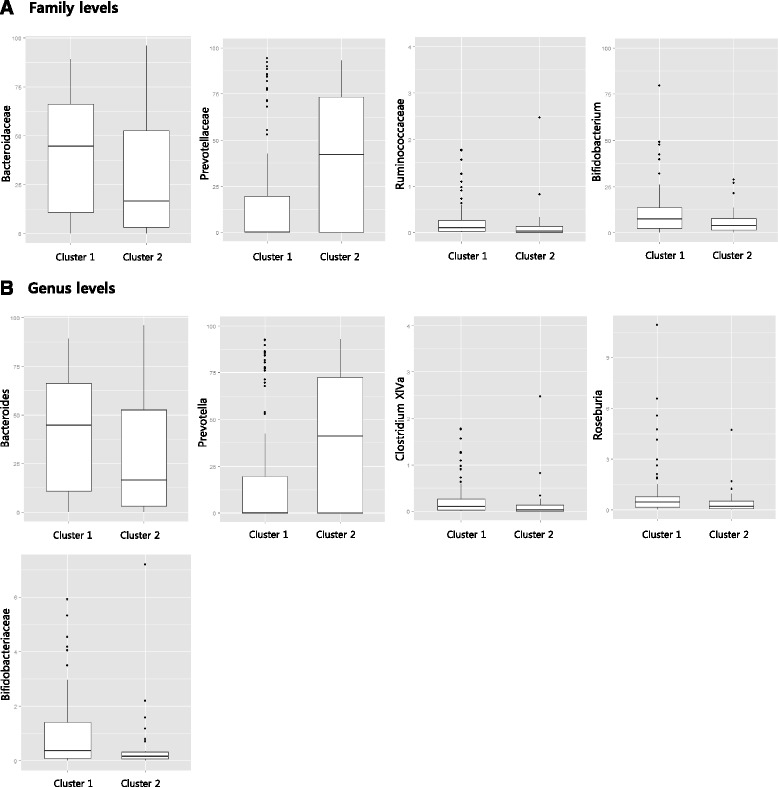
Association of clusters with abundant microbiota in family and genus levels. The Wilcoxon rank-sum test was used to assess the association of clusters with microbiota (P < 0.05). Boxes represent the interquartile range (IQR) between the first and third quartiles with a line at the median

As reported previously [4], we calculated the number of OTUs to examine the diversity of the gut microbiota based on an average of 8,846 reads which covered each sample. The mean number of OTUs was 356 ± 140 (range, 105–876) and the number of OTUs between normal and obese individuals (Mann–Whitney *U* test, *p* = 0.072) had no significant difference. Also, there was no obvious difference between these samples when the alpha-diversity (Shannon Index) values between normal and obese samples were compared (OTUs, average normal 6.94 ± 0.49, obese 6.98 ± 0.59). In addition, PCA result of beta diversity did not show differential pattern between the normal and obese samples (data not shown). Table 4 and Fig. 1 show the average fecal microbiota compositions of the dietary pattern groups according to taxonomic rank (*P* < 0.05). Notably, there was a marked difference in the abundance of the genus *Bifidobacterium* between Cluster 1 and Cluster 2 (*P* = 0.011). The proportion of *Bifidobacterium* in Cluster 1 was 0.26%, compared with 0.18% in Cluster 2. This trend was also evident for the family *Bifidobacteriaceae* and the phylum *Actinobacteria* (0.26 and 0.18%, and 0.35 and 0.25% respectively). We also found significant differences in the proportions of the genera *Bacteroides*, *Prevotella*, *Clostridium XlVa*, and *Roseburia* (*P* = 0.046, 0.015, 0.026, and 0.046, respectively) and the families *Bacteroidaceae*, *Prevotellaceae*, and *Ruminococcaceae* (*P* = 0.046, 0.047, and 0.047, respectively) according to dietary pattern group. Cluster 1 had a higher proportion of *Bacteroides* (*Bacteroidaceae*), *Clostridium XlVa*, and *Roseburia*, but a lower proportion of *Prevotella* (*Prevotellaceae*) compared with Cluster 2. However, there were no significant difference in above-mentioned genera and families by the dietary patterns after adjustment of FDR.

**Table 4:** List of taxa showing different abundance between dietary patten groups

	Traditional diet	Modified Western diet	P-value^a^ (unadjusted)	P-value (FDR adjusted)
	(median)	(median)		
Genus levels				
*Bacteroides*	44.53	16.58	0.046	0.110
*Prevotella*	0.03	41.26	0.015	0.090
*Clostridium XlVa*	0.45	0.22	0.026	0.104
*Roseburia*	0.37	0.15	0.046	0.110
*Bifidobacterium*	0.10	0.02	0.011	0.090
Family levels				
*Bacteroidaceae*	44.53	16.58	0.046	0.129
*Prevotellaceae*	0.20	41.98	0.047	0.129
*Ruminococcaceae*	7.50	3.86	0.047	0.129
*Bifidobacteriaceae*	0.10	0.03	0.012	0.129
Phylum levels				
*Actinobacteria*	0.14	0.06	0.020	0.100

^a^ *P*-value was obtained from the Wilcoxon rank-sum test

### Risk of obesity and metabolic syndrome

We evaluated the association between fecal microbiota and metabolic disease/or disorders using multivariate-adjusted ORs (Table 5). Because of the absence of normal weight subjects with MS, the normal weight group without MS was used as the reference and compared with obesity group with and without MS in this study. Lower proportion of *Bacteroides* (*Bacteroidaceae*) or higher proportion of *Prevotella* (*Prevotellaceae*) had significantly higher risk effect on obesity and the risk tended to increase when obesity subjects had with MS. We also found that lower proportion of *Ruminococcaceae* was associated with a higher risk of obesity and had a tendency slightly to increase risk in obesity with MS although there was no significant effect.

**Table 5:** Association between fecal microbiota and risk of obesity with and without metabolic syndrome

Taxa	Obesity without MS		*p*	Obesity with MS		*p*
	OR	(95% CI)		OR	(95% CI)	
Genus levels						
*Bacteroides*	0.973	(0.957-0.990)	0.002	0.965	(0.946-0.985)	0.001
*Prevotella*	1.018	(1.004-1.032)	0.011	1.025	(1.009-1.040)	0.002
*Clostridium XlVa*	0.692	(0.391-1.227)	0.208	1.088	(0.822-1.440)	0.556
*Roseburia*	1.018	(0.740-1.400)	0.915	0.844	(0.545-1.308)	0.449
*Bifidobacterium*	1.331	(0.510-3.474)	0.559	0.400	(0.074-2.163)	0.287
Family levels						
*Bacteroidaceae*	0.973	(0.957-0.990)	0.002	0.965	(0.946-0.985)	0.001
*Prevotellaceae*	1.018	(1.004-1.032)	0.011	1.024	(1.009-1.040)	0.002
*Ruminococcaceae*	0.973	0.885-0.994)	0.030	0.973	(0.935-1.013)	0.184
*Bifidobacteriaceae*	1.330	(0.509-3.471)	0.560	0.400	(0.074-2.161)	0.287
Phylum levels						
*Actinobacteria*	1.171	(0.561-2.446)	0.674	0.646	(0.220-1.895)	0.426

*P* value of logistic regression with adjustment for age and sex

### Associations of microbiota composition with dietary intake

**Fig. 2:**
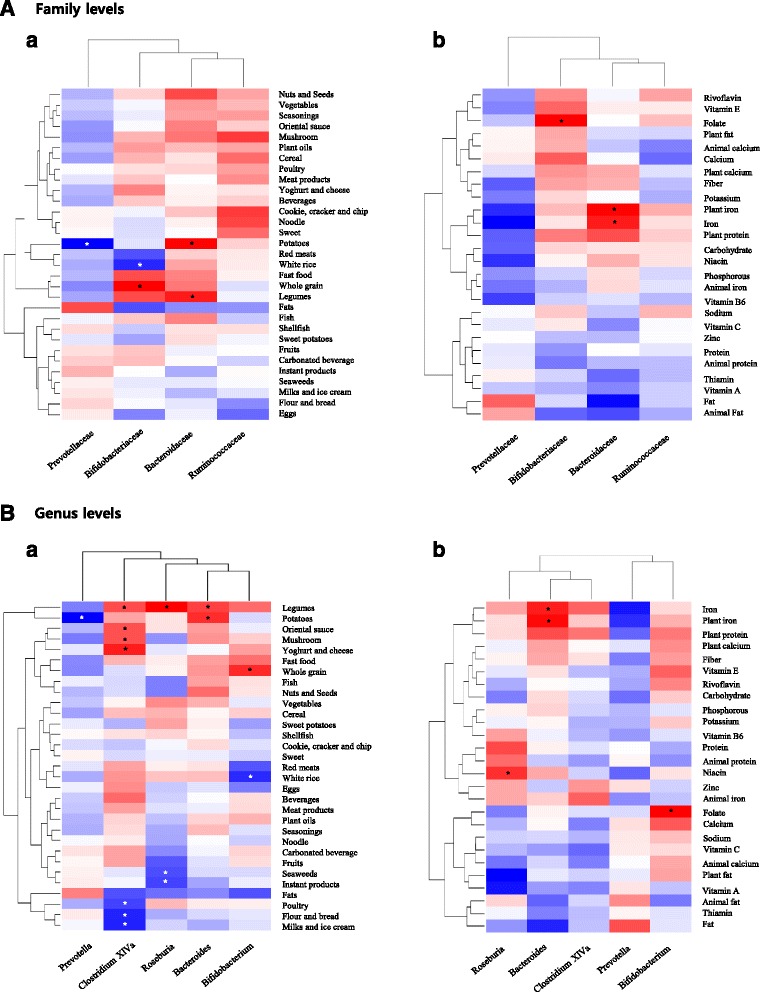
Spearman’s correlations between dietary intakes and microbial taxa. Columns correspond to bacterial taxa showing a significant difference in abundance according to dietary pattern group; rows correspond to **a** age, sex BMI-adjusted intakes of food items and **b** age, sex, energy and BMI-adjusted nutrients intakes (* adj *P* < 0.05) Columns and rows are cluster by hierarchical method

Fig. 2 shows a heat map of Spearman’s correlations between dietary intake and microbial taxa. We considered only five genera and four families that exhibited a significant difference in abundance according to dietary pattern group. After adjustment age, sex, and BMI, the high proportion of *Bifidobacteriaceae* and *Bifidobacterium* were associated with high intakes of whole grain and low white rice consumption. The *prevotellaceae* and *prevotella* were negatively associated with potatoes, whereas *Bacteroidaceae* and *Bacteroides* showed the opposite association. The microbiota composition positively associated with legumes were *Bacteroides* (*Bacteroidaceae*), *Roseburia*, and *Clostridium XlVa*. In addition, *Clostridium XlVa* was associated with high intakes of legumes and fermented foods, such as yoghurt, cheese, and oriental sauce, and low intake of milk, poultry, and Flour. *Roseburia* was inversely associated with seaweeds and instant products.

We next examined the association between microbiota composition and nutrient intake. *Bacteroides* (*Bacteroidaceae*) was positively associated with iron, especially plant iron. We also found several positive associations, e.g., folate with *Bifidobacterium* (*Bifidobacteriaceae*) and niacin with *Roseburia*.

## Discussion

There has been increasing interest in analyzing dietary factors associated with chronic disease. The analysis of dietary patterns has advantages over nutrient-based or food-group-based dietary assessment approaches because people consume foods in the form of meals, which are combinations of various foods and nutrients. Therefore, we analyzed dietary patterns in Korean adolescents using a combination of factor and cluster methods and compared the fecal microbiota and host phenotype across these dietary pattern groups.

In this study, we identified two major dietary patterns: traditional (Korean) and modified Western diets. The traditional diets were characterized as comprising predominantly plant-based and fermented foods because they included higher proportions of oriental sauce, sweet potatoes, vegetables, seaweeds, and whole grains, which are naturally high in fiber and undergo minimal processing. In contrast, the modified Western diets were characterized as comprising animal-based foods with intake of red meats, fishes, oils, and fats. Although the two dietary patterns were statistically independent as determined by the orthogonal rotation procedure, it would be possible for one individual to have high or low scores for both dietary patterns simultaneously [29]. In our study, white rice had high loadings in both the traditional and modified Western diets, as rice is the staple in the Korean diet.

Many studies have attempted to identify an association between dietary patterns and the composition of the gut microbiota. Here, we examined bacterial composition at the genus, family, and phylum levels according to dietary pattern group. We found that adolescents who consumed traditional diets showed a higher proportion of *Bacteroides* (*Bacteroidaceae*), *Clostridium XlVa*, *Roseburia*, *Bifidobacterium* (*Bifidobacteriaceae–Actinobacteria*) and *Ruminococcaceae* and a lower proportion of *Prevotella* (*Prevotellaceae*), whereas inverse associations were found for those who consumed modified Western diets. In our study, traditional diets comprised predominantly plant-based and fermented foods, which are low in fat and rich in carbohydrate, plant protein, vitamin, mineral, and fiber; in contrast, modified Western diets comprised greater proportions of animal-based foods and were high in fat and low in fiber. In support, previous studies have reported that vegetarian diets were associated with *Bacteroides* [11], and fat-restricted diets, together with higher carbohydrate intake, were linked to increased proportions of *Bacteroides* and *Bifidobacterium* [30]. However, Cani et al. reported a reduction in *Clostridium cluster XIVa* and lower *Bifidobacterium* and *Bacteroides* levels [31] and Kim et al. found the enrichment of *Ruminococcaceae* [32] in mice fed a high-fat diet.

Meanwhile, Wu et al. [8] reported that diets high in animal protein and fats, similar to a Westernized diet, were associated with a *Bacteroides* enterotype. By contrast, the *Prevotella* enterotype was associated with diets high in carbohydrates that contained more plant-based foods. Similarly, De Filippo et al. [7] found that children in rural Africa showed a higher abundance of *Prevotella*, whereas European children had a higher abundance of *Bacteroides.* The authors speculated that the abundance of *Prevotella* in rural African children was a consequence of their higher fiber intake because the traditional rural African diet is primarily vegetarian, being low in fat and animal protein and rich in starch, fiber, and polysaccharides. In contrast, the Western diet of the European children was high in animal protein, sugar, starch, and fat, and low in fiber. We assume that the inconsistencies with our findings are due to the complex relationships among genetic, geographical, environmental, technical, and/or clinical factors. The diet of people living in Korea, even those who consuming modified Western diets, is usually rather low in fat and provides a high amount of carbohydrate and fiber compared with the diet of people living in Western countries. Several studies showed that the microbiota composition clustered according to country in spite of a cultural area similar to the Western diet and quantity of *Prevotella* was greater in people from geographical regions where plant-based dietary pattern dominated [6, 33]. Furthermore, some reports suggested that the genus *Prevotella* was underrepresented in Americans probably due to discriminatory taxon, which is the enrichment in Prevotella in African children compared with European children, in Africans compared with African Americans, in the Hadza hunter-gatherers (from Tanzania) compared with Italian people, and in Succinivibrio and Treponema in several African populations [6, 34]. Therefore, more integrated approaches are needed to enhance our understanding of these complex associations.

It has been reported that the gut microbial communities of Koreans individuals differed from those of US, Japanese, and Chinese subject, but tended to vary less between individual Koreans, suggesting that diet type affects the gut microbiota [35]. They also found the core gut microbiota in Korean individuals and many of these are related to butyrate-producing bacteria. Koreans have high intakes of carbohydrate and fiber [36, 37], which are related to production of short-chain fatty acids (SCFA) such as acetate, propionate, and butyrate. SCFA, especially butyrate, have been suggested as important in maintaining gut health. Most subjects in our study were in the traditional diet group (73.2%), which was associated with a greater proportion of butyrate-producing bacteria, including *Bacteroides*, *Clostridium XlVa*, and *Roseburia.* Although we could not evaluate the subsequent effects of the gut microbiota related to dietary pattern and fecal metabolite in the current study, we believe that this should be taken into consideration in future in-depth studies.

Several studies have reported an association of major dietary patterns with obesity and metabolic disease. In this study, we found that subjects who consumed a modified Western diet had a higher prevalence of obesity and metabolic syndrome than those who consumed a traditional diet. In support of this, previous cross-sectional studies reported that Western dietary patterns were positively associated with obesity [38, 39]. Two large prospective cohort studies, the Nurses’ Health Study and the Health Professionals Follow-up Study, also reported that adoption of a Western dietary pattern was associated with greater weight gain [40], whereas reduction of weight gain was linked to diets rich in plant-based foods [41]. Furthermore, these studies reported that a Western diet was significantly related to metabolic diseases such as CVD [42, 43] and T2D [44, 45] and to all-cause mortality [43]. The Western dietary pattern was associated with increased risks of these diseases, whereas a prudent dietary pattern, diet high in unprocessed foods and fiber and low in salt, fat, and sugar, was associated with beneficial effects on host health [42–45]. In our study, we found that several important components of metabolic disorder were associated with dietary pattern. Subjects who consumed a modified Western diet had higher levels of liver enzymes, TC and TG, hs-CRP, insulin, and HOMA-IR than those who consumed a traditional diet, and these values were associated with increased risk of CVD and T2D. Also, we investigated that high consumption of whole grain and fiber or low consumption of fats is positively associated with *Bacteroides,* but not *Prevotella* (data not shown). However, the association disappeared after adjustment for BMI, because most obese adolescents consumed modified Western diet characterized with low intake of fiber and high intake of fat in the current study. This indicates the effects of fiber and fat intakes on gut microbiota composition including *Bacteroides* and *Prevotella*, which is associated with the pathogenesis of metabolic disease.

Many studies have attempted to identify an association between the composition of the gut microbiota and metabolic disease. In this study, we found that increased in *prevotella* (*Prevotellaceae*) and decreased in *Bacteroides* (*Bacteroidaceae*) and *Ruminococcaceae* had a higher risk of obesity. Theses tendency increased when they had with MS in genera and families of *prevotella* (*Prevotellaceae*) and *Bacteroides* (*Bacteroidaceae*). However, the evidence for an association between gut microbiota and metabolic disease is scarce. Recent studies suggested a potential role for diet in promoting a gut microbiome associated with the pathogenesis of metabolic disease. Koeth et al. [46, 47] found that microbial metabolism of choline, phosphatidylcholine and L-carnitine resulted in production of trimethylamine (TMA), which is further metabolized to the proatherogenic species, trimethylamin-N-oside (TMAO). Together, these findings suggested that individuals adhering to an omnivorous diet have higher fasting TMAO concentrations and produce more TMAO than do vegans or vegetarians after ingestion of carnitine through a microbiota-dependent mechanism. Furthermore, a correlation analysis of the fecal microbiota and plasma TMAO levels indicated that subjects with a *Prevotella* enterotype had significantly higher plasma TMAO concentrations than did *Bacteroides* enterotype subjects [47]. Interestingly, our study showed that subjects who consumed a modified Western diet had a greater abundance of *Prevotella*. Additionally, we recently reported that an increased *Prevotella* population was associated with increased TC, TG, and hs-crp levels, but negatively associated with HDL-C [4].

Due to the fact that QIAamp DNA Stool kit based on enzymatic DNA-extraction has the lower diversity of fecal microbiota comparing other DNA extraction kits [48] and is a insufficient method to lyse cell wall efficiently in detecting genes of various Gram-positive bacteria [49], caution must be used when interpreting these findings as regards to the general adolescent fecal microbiota. Additionally, short-term dietary data on 3-day food diaries maintained for three consecutive days (2 weekdays and 1 weekend day) were obtained from self-reported questionnaires which are not representative of the long-term dietary pattern of the subjects and may have resulted in an estimation bias. Finally, our study was cross-sectional in design. In this context, further studies with different methods using a prospective design and measurements before and after interventions are needed to determine core microbiota, the effects of dietary patterns, and the causal relationships among microbiota, dietary patterns, metabolic markers, and disease.

Nevertheless, we found that the proportions of the genera *Bacteroides*, *Prevotella, Clostridium XlVa*, *Roseburia*, and *Bifidobacterium* were markedly different in subjects who consumed traditional compared with those who consumed modified Western diets. Furthermore, a traditional (Korean) dietary pattern was associated with reduced risk of metabolic disease, whereas opposite associations were found for a modified Western dietary pattern. These findings suggest that dietary habits affect both the gut microbiota composition and host health.

## Conclusions

We found that traditional dietary patterns were associated with higher proportions of *Bacteroides* (*Bacteroidaceae*) and *Bifidobacterium* (*Bifidobacteriaceae-Actinobacteria*) and a lower proportion of *Prevotella* (*Prevotellaceae*) relative to modified Western dietary patterns. Specifically, the proportion of *Bacteroides* (*Bacteroidaceae*) was associated with intake of plant-based nutrients such as fiber; however, that of *Prevotella* (*Prevotellaceae*) was negatively associated with these factors. Also, we found that *prevotella* (*Prevotellaceae*) increased and *Bacteroides* (*Bacteroidaceae*) and *Ruminococcaceae* decreased in higher risk group of obesity. The risk had a tendency to increase in obesity subjects with MS in genera of *prevotella* and *Bacteroides* and families of *Prevotellaceae* and *Bacteroidaceae*. We also found that the traditional dietary pattern was negatively associated with general and central adiposity and levels of clinical biomarkers, including AST, ALT, total cholesterol, triglyceride, hs-CRP, insulin, and HOMA-IR, whereas the positive associations were found for a modified Western dietary pattern. These findings suggest that dietary habits affect both the gut microbiota composition and host health.

## Additional file

Additional file 1: Table S1. Food grouping used in the dietary pattern analysis. **Table S2.** Nutrient intakes of subjects across dietary pattern groups. (DOCX 17 kb)

## Abbreviations

ALT: Alalnine aminotransferase
AST: Aspartate aminotransferase
BMI: Body mass index
CVD: Cardiovascular disease
HDL-C: High-density lipoprotein-cholesterol
HOMA-IR: Homeostasis model assessment of insulin resistance
hs-crp: High-sensitivity C-reactive protein
IR: Insulin resistance
MS: Metabolic syndrome
OTUs: Operational taxonomic units
T2D: Type 2 diabetes
TC: Total cholesterol
TG: Triglyceride
TMAO: Trimethylamin-N-oside
