# Supplementary material for: Association of dietary patterns with the fecal microbiota in Korean adolescents
Source: BMC Nutr. 2017 Mar 4;3:20. doi: 10.1186/s40795-016-0125-z (PMC7050889; doi:10.1186/s40795-016-0125-z)
Supplement: Supplementary file 1 — Food grouping used in the dietary pattern analysis. Table S2. Nutrient intakes of subjects across dietary pattern groups. (DOCX 17 kb) [file 40795_2016_125_MOESM1_ESM.docx]

**Table S1.** Food grouping used in the dietary pattern analysis

| Food group | Food items |
| --- | --- |
| White rice | White rice |
| Whole grains | Brown rice, barley, millet etc. |
| Noodle | Noodles |
| Cereal | Breakfast cereals |
| Flour and bread | Wheat flour, breads, doughnuts, cakes. |
| Cookie, cracker and chip | Cookies, cracker, snack, biscuits, chip |
| Instant products | Instant noodle, can products |
| Fast Food | Pizza, hamburger, sandwich, french fried potato |
| Potatoes | Potatoes and starch |
| Sweet potatoes | Sweet potatoes |
| Sweet | Sugar, honey, starch syrup, candy |
| Legumes | Beans |
| Nuts and Seeds | Peanuts, almonds, pine nut, chestnut, sesame seeds |
| Vegetables | Vegetables |
| Mushroom | Mushroom |
| Fruits | Fruits |
| Red meats | Beef, pork |
| Poultry | Chicken, turkey |
| Meat products | Ham, sausage, meatball, bacon (processed) |
| Eggs | Eggs |
| Fish | Salted fish, cuttlefish, canned tuna |
| Shellfish | Shellfish |
| Seaweeds | Seaweeds |
| Milks and ice cream | Milk, ice cream |
| Yoghurt and cheese | Yoghurt and cheese |
| Plant oils | Sesame oil, soybean oil, corn oil |
| Fats | Animal fats, magarine, butter, mayonnaise |
| Carbonated beverage | Soft drink |
| Beverages | Other drink |
| Oriental sauce | Soy sauce, red pepper, miso |
| Seasonings | Other seasoning |

**Table S2.** Nutrient intakes of subjects across dietary pattern groups

|  | **Traditional diet**  **(n=82)** | **Modified Western diet**  **(n=30)** | ***P*** ^a^ |
| --- | --- | --- | --- |
| Energy (kcal) | 1710.4 ± 352.8 | 1687.7 ± 509.8 | 0.6337 |
| C: P: F (%)^b^ | 67.2: 19.1: 13.7 | 63.7: 20.3: 16.0 |  |
| Carbohydrate (g) | 247.0 ± 50.1 | 229.3 ± 79.2 | 0.0054 |
| Protein (g) | 70.7 ± 18.4 | 70.1 ± 20.6 | 0.9153 |
| Animal Protein | 37.3 ± 14.4 | 41.2 ± 14.8 | 0.0582 |
| Plant Protein | 33.4 ± 8.7 | 28.8 ± 10.3 | 0.0018 |
| Fat (g) | 50.8 ± 15.8 | 55.3 ± 18.4 | 0.0138 |
| Animal Fat | 28.5 ± 13.8 | 30.5 ± 18.5 | 0.3225 |
| Plant Fat | 22.3 ± 9.2 | 24.8 ± 11.1 | 0.1312 |
| Vitamin A (ugRE) | 746.5 ± 326.2 | 673.7 ± 323.4 | 0.2886 |
| Thiamin (mg) | 1.2 ± 0.4 | 1.3 ± 0.6 | 0.3051 |
| Rivoflavin (mg) | 1.1 ± 0.3 | 1.1 ± 0.4 | 0.3717 |
| Niacin (mg) | 16.4 ± 5.0 | 16.7 ± 6.8 | 0.6603 |
| Vitamin B_6_ (mg) | 1.9 ± 0.6 | 1.7 ± 0.6 | 0.0101 |
| Folate (ug) | 217.2 ± 79.7 | 173.4 ± 72.2 | 0.0011 |
| Vitamin C (mg) | 99.0 ± 57.0 | 57.2 ± 31.9 | <0.0001 |
| Vitamin E (mg) | 13.4 ± 5.1 | 14.2 ± 6.9 | 0.3689 |
| Calcium (mg) | 487.5 ± 175.0 | 345.8 ± 195.7 | <0.0001 |
| Animal Calcium | 207.1 ± 128.3 | 147.5 ± 131.5 | 0.0389 |
| Plant Calcium | 280.4 ± 95.7 | 198.3 ± 89.6 | <0.0001 |
| Phosphorus (mg) | 953.9 ± 270.5 | 849.3 ± 280.6 | 0.004 |
| Sodium (mg) | 4164.2 ± 1249.5 | 3344.3 ± 1201.4 | <0.0001 |
| Potassium (mg) | 2393.2 ± 813.9 | 1991.3 ± 800.6 | 0.0017 |
| Iron (mg) | 12.4 ± 3.6 | 10.6 ± 3.4 | 0.0017 |
| Animal Iron | 3.4 ± 1.7 | 3.2 ± 1.0 | 0.6297 |
| Plant Iron | 9.0 ± 2.8 | 7.2 ± 2.8 | 0.0002 |
| Zinc (mg) | 8.6 ± 2.3 | 8.1 ± 2.4 | 0.1941 |
| Fiber (g) | 17.9 ± 5.5 | 13.5 ± 5.2 | <0.0001 |

Data are expressed as the mean±S.D.

^a^ *P* value was calculated by generlized linear regression analysis with age, sex and energy intakes.

^b^ C:P:F (%).; Percentages of energy from carbohydrate: protein: fat
